# A Rare Occurrence of Isolated Endogenous Escherichia coli Panophthalmitis: A Case Report

**DOI:** 10.7759/cureus.47059

**Published:** 2023-10-15

**Authors:** Nadhirah Ahmad Fauzi, Abdul-Hadi Rosli, Aidila Jesmin Jabbari

**Affiliations:** 1 Ophthalmology, Kulliyyah of Medicine, International Islamic University Malaysia, Kuantan, MYS

**Keywords:** diabetes mellitus, intravitreal antibiotic administration, ocular infection, orbital cellulitis, escherichia coli, panophthalmitis

## Abstract

Panophthalmitis is a severe ocular condition that can lead to devastating outcomes, such as evisceration, if not promptly treated. It typically originates endogenously, with urinary tract infection being a common primary source of infection. This report describes a rare case of a 61-year-old Malay patient with left eye panophthalmitis. Ocular culture revealed *Escherichia coli*, while systemic septic workup yielded negative results. Due to the severity of the ocular condition at presentation and the disproportionate level of pain the patient had, an immunocompromised state was suspected and later the diagnosis of diabetes mellitus was confirmed via laboratory investigation. Despite the delay in presentation, which hindered early intervention, the patient’s eyeball was successfully salvaged through a treatment regimen involving three injections of intravitreal antibiotic administered at 48 to 72-hour intervals and a complete course of intravenous antibiotics. This case report highlights the importance of prompt treatment to salvage an eye from evisceration in the case of panophthalmitis.

## Introduction

Panophthalmitis is an infectious process caused by a pyogenic organism that encompasses all structures of the globe along with surrounding orbital and periorbital structures [1]. This disease develops rapidly and the prognosis is poor [2]. It results from pyogenic organism multiplication within the eye after bacteria cross the blood-ocular barrier during sepsis or bacteremia [3]. The immunocompromised state of the patient is a major risk factor for its occurrence [4]. The management of patients with panophthalmitis remains challenging with most patients ending up with evisceration [5].

## Case presentation

**Figure 1:**
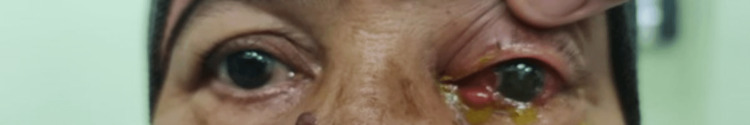
Limited facial photograph showing left eye proptosis, ptosis, and severe conjunctival chemosis.

**Figure 2:**
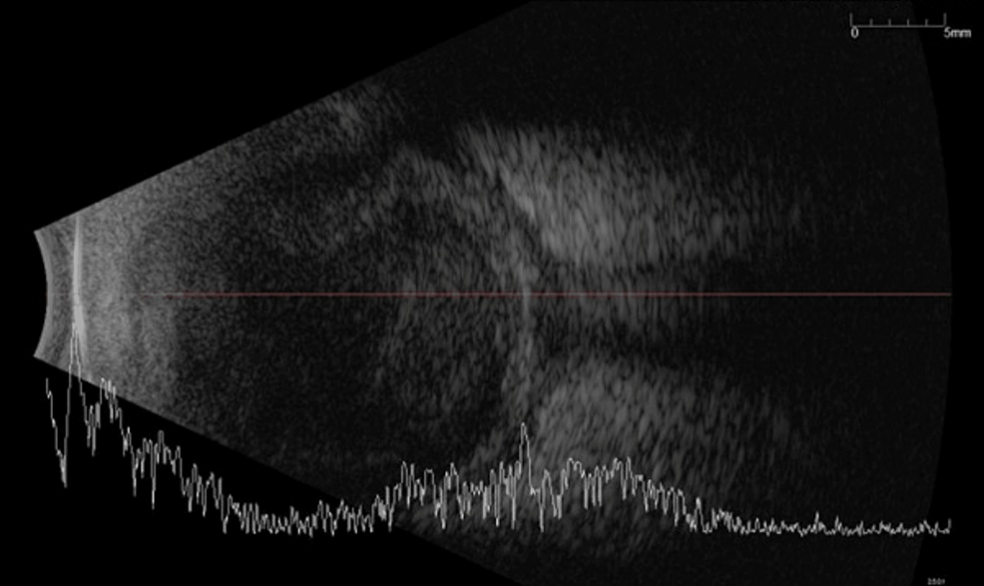
Ultrasound B-scan showing dense vitreous loculation and diffuse scleral thickening (T-sign).

A 61-year-old Malay lady with no known medical illnesses presented with a one-week history of progressive left periorbital swelling and redness. It was associated with blurring of vision, discomfort, and eye discharge. The patient did not complain of severe eye pain. One week before the onset of ocular symptoms, the patient had a five-day history of low-grade fever and urinary frequency. The patient was seen by a general practitioner for the symptoms of a urinary tract infection and treated with oral moxifloxacin. The patient denied any history of ocular trauma or insect bite. On presentation, the patient had no fever, no cough or runny nose, no dysuria or urinary frequency, and no suprapubic pain. Clinically, there was no documented temperature, no abdominal tenderness, no suprapubic tenderness, and the renal punch was negative. On examination of the eye, visual acuity of the left eye was a perception of light, intraocular pressure (IOP) was 54 mmHg, there was restricted extraocular movement in all gazes, and there was positive relative afferent pupillary defect (RAPD). Anterior-segment examination showed proptosis, ptosis, severe chemosis of the conjunctiva, generalized corneal edema, a shallow anterior chamber with the presence of dense fibrin covering the pupil, hypopyon, and cataractous lens (Figure 1). Ultrasound B-scan of the left eye showed dense vitreous loculation and diffuse scleral thickening or T-sign (Figure 2). Her right eye was otherwise normal with a visual acuity of 6/6.

**Figure 3:**
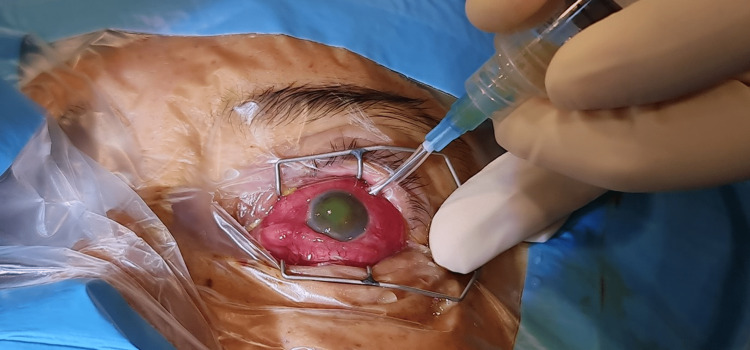
Intravitreal tapping performed under aseptic technique. The cornea appeared hazy due to high intraocular pressure and infection.

**Figure 4:**
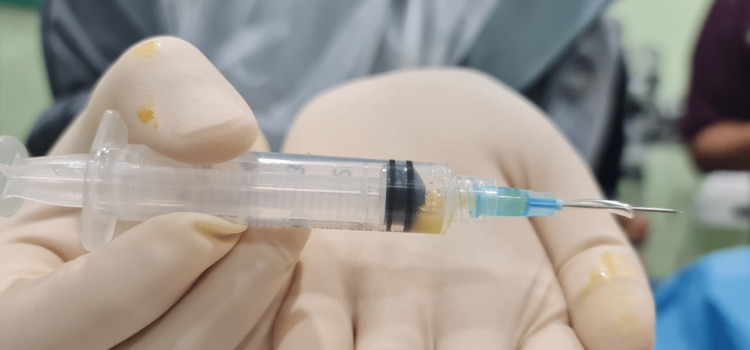
Vitreous tap showing thick purulent yellowish vitreous fluid.

The diagnosis of left eye panophthalmitis was made and immediate intravitreal tapping yielded a thick purulent yellowish vitreous fluid (Figures 3, 4). The fluid was sent for culture and sensitivity testing.

**Figure 5:**
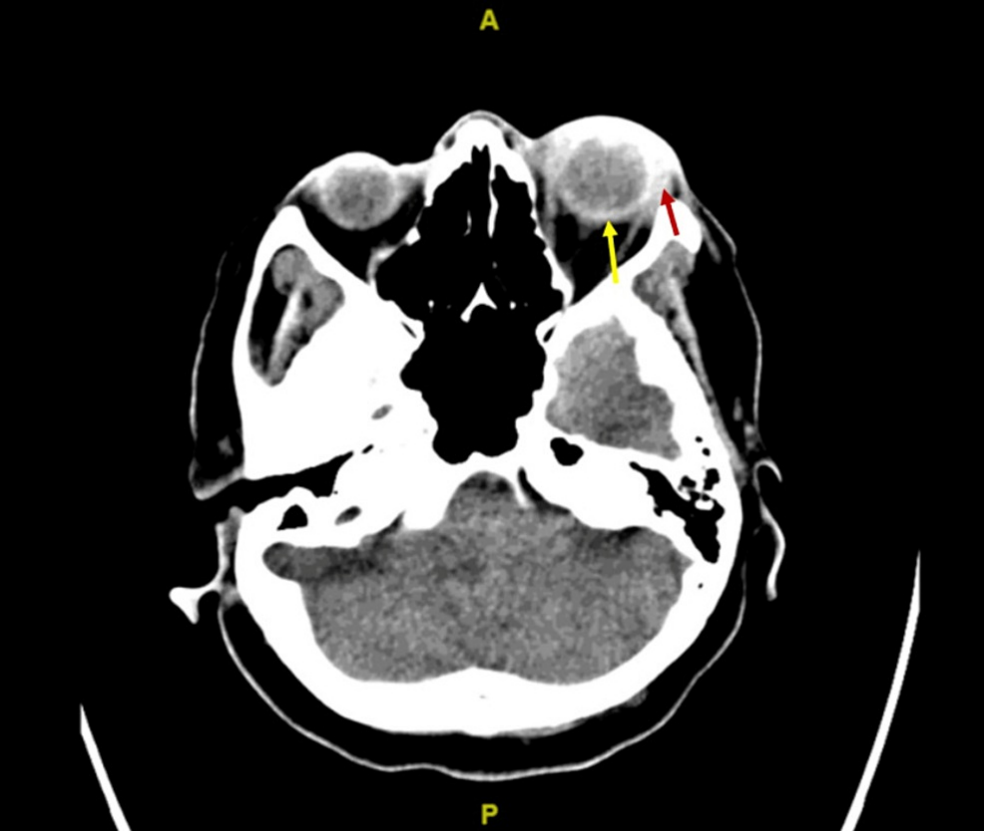
CT scan of the brain and orbit showing left globe proptosis, diffuse thickening of the sclera, choroidal enhancement (yellow arrow), and denser appearance of aqueous and vitreous humor of the left globe, as well as extensive left periorbital fat stranding (red arrow).

The diagnosis was supported by a CT scan of the brain and orbit which showed left globe proptosis, diffuse thickening of the sclera, choroidal enhancement, and denser appearance of aqueous and vitreous humor of the left globe, as well as extensive left periorbital fat stranding (Figure 5).

In view of the severity of infection at presentation, an immunocompromised state was suspected and later the diagnosis of diabetes mellitus was confirmed via laboratory investigation of HbA1C level at 7.8%. Other series of investigations sent were blood culture and sensitivity, UFEME, urine culture and sensitivity, and ultrasound of the abdomen to look for possible sources of infection. However, all results were negative. Referrals were also made to the ENT team to look for possible ascending infection from the sinuses which revealed no sinusitis and absence of bony erosions to suggest fungal infection. The vitreous fluid sent for culture and sensitivity later came back positive for *Escherichia coli*.

She was initially treated with an intravitreal injection of vancomycin 1 mg/0.1 mL and ceftazidine 2.2 mg/0.1 mL. After receiving the results of the gram stain, which came back as gram-negative bacillus, intravitreal amikacin 0.4 mg/0.1 mL was added in the second and third intravitreal injections. A total of three intravitreal injections were administered at an interval of 48 to 72 hours. In addition, she received topical antibiotics which included moxifloxacin 0.5%, gentamicin 0.9%, and ceftazidime 5%. Systemically, she received a complete course of intravenous ceftriaxone 1 g daily, gentamicin 240 mg daily, and metronidazole 500 mg three times daily for two weeks. Her high IOP was managed with a maximum of four IOP-lowering medications including travaprost 0.004%, timolol 0.5%, brinzolamide 1%, and brimonidine 0.2%. Following treatment, the patient showed significant improvement in the left eye proptosis with the resolution of conjunctival chemosis, hypopyon, and anterior chamber fibrin. Even though the final visual acuity was no light perception, we were able to avoid evisceration in this patient.

## Discussion

Panophthalmitis is a severe ocular and orbital condition with poor prognostication as it may necessitate evisceration [3]. The most common cause of panophthalmitis is post-traumatic endophthalmitis followed by postoperative endophthalmitis after cataract surgery [6]. It can also be caused by endogenous spread by septicemia or sources within the body. Thus, in the absence of trauma or surgical wounds, investigations must be directed toward looking for the primary source of infection.

In 90% of the cases where infectious extraocular focus can be found, the sources may include tooth abscess, pneumonia, endocarditis, liver abscess, urinary tract infection, and bacterial meningitis. However, in around 10-20% of cases, the source could not be identified [2]. Bacterial causes are responsible for half of endogenous endophthalmitis and are divided into gram-positive and gram-negative organisms [7-10]. *Escherichia coli* from urinary tract infection is the second leading cause. In this patient, the urine culture came back negative; however, the patient may have been partially or fully treated for urinary tract infection as she was already treated with oral antibiotics for one week when she had an episode of dysuria a week before.

Panophthalmitis is more commonly seen in immunocompromised patients with diabetes being the most common association [8,9]. Other risk factors for immunocompromised states include HIV/AIDS, systemic malignancy, sickle cell anemia, neonates, and patients on immunosuppressive treatment [3].

Regarding treatment goals, in this case, the patient already presented with a positive RAPD indicating an irreversible insult that had been done to the optic nerve. Another predictor of poor visual outcome in this patient was light perception only vision at presentation. Thus, visual recovery was not the primary goal when treating this patient but instead aimed toward globe preservation. Globe preservation is important for patients as it provides better cosmetic and physiological results and improves the psychological status of the patients.

Immediate and timely management of panophthalmitis not only aims at preserving anatomical integrity but also prevents the upward spread of infection to the brain and prevents systemic spread which can be life-threatening [4]. Surgical management is pursued after medical options have been exhausted and they include evisceration, enucleation, and pars plana vitrectomy. Pars plana vitrectomy plays a role in removing the locus of infection as well as removing the toxic debris from the vitreous cavity [11]. However, the type of surgical options are tailored depending on the clinical condition of each patient.

Intravitreal tapping is beneficial not only to obtain samples for microbial testing but also to mechanically remove the causative agent, while intravitreal antibiotic injection aims at delivering medications directly at the locus of infection, which in this case was the vitreous cavity [12]. Reinjection of intravitreal antibiotic is considered when resolution of infection is not achieved or fails to stabilize for more than 48 hours. Often, 36 hours after treatment, culture results would be available, thus, reinjection with suitable antibiotics can be performed. In this case, three injections of intravitreal antibiotics were needed to achieve improvement and stabilization of infection.

## Conclusions

This case highlights a severe presentation of left eye panophthalmitis in an immunocompromised patient with diabetes mellitus. Prompt diagnosis and management with intravitreal and systemic antibiotics led to the resolution of the infection. As this patient presented with predictors of poor visual outcomes that included a positive RAPD and light perception-only vision, the avoidance of evisceration was considered a positive outcome.
